# Supplementary material for: Effect of Online Home-Based Training on Functional Capacity and Strength in Two CKD Patients: A Case Study
Source: Healthcare (Basel). 2022 Mar 18;10(3):572. doi: 10.3390/healthcare10030572 (PMC8951501; doi:10.3390/healthcare10030572)
Supplement: Supplementary file 1 [file healthcare-10-00572-s001.zip › healthcare-1603033-supplementary.pdf]

Table S1: Schedule exercises' example of the online training program

SCHEDULE EXERCISES	
**WARM-UP**	
**DURATION:** 15′	**AIM:** Mobility, Proprioceptive, Balance Ex.<br>**EQUIPMENT:** Wands, Chair
**EXERCISE**	**Repetitions**
Circumduction of Shoulder – Calf Raise	8-10
Dynamic Hip Mobility on Sagittal and Frontal Plane	8-10 + 8-10
Circumduction of Arms – Half Squat	8-10
Flex-Extensions of Arms + Wands	8-10
Walking on Heel, Forefoot, Tandem	1′-2′
Monopodalic Balance + Chair and Dynamic Balance Y-Balance	10″-30″

STRENGTH PHASE				
**DURATION:** 20′- 30′	**MUSCLE GROUPS:** Total body, Core<br>**EQUIPMENT:** Thera Band (TB), Mat			
**CIRCUIT (1) x 3 set**	**Week 1**	**Week 2**	**Week 3**	**Week 4**
Sumo squat + TB	10	10	15	15
Bent over row + TB	10	10	15	15
Romanian Dead Lift + TB	10	10	15	15
Rest	60′′	45′′	45′′	30′′
**CIRCUIT (2) x 3 set**	**Week 1**	**Week 2**	**Week 3**	**Week 4**
Inclined Push up	10	10	15	15
Backward Lunges – Lateral Raise + TB	8+8	8+8	10+10	10+10
Inclined walking plank Into Squat	10	10	15	15
Rest	60′′	45′′	45′′	30′′

AEROBIC PHASE	
**DURATION:** 10′-15′	**INTENSITY:** ≃65-70% of HRR<br>**MUSIC:** ≃128 bpm
**STEPS**	**Repetitions**
March in place with Arms	3x8
Sidestep	3x8
Knee Up	3x8
Leg Curl	3x8
Kick and Punge	3x8
Lounges	3x8
V-Step	3x8
March in place with Arms	3x8

COOL-DOWN	
**DURATION:** 5′-10′	**AIM:** Stretching of all muscle groups involved in the protocol
